# Prognostic Factors Affecting Graft Survival in Patients Undergoing Penetrating Keratoplasty for Infectious Keratitis

**DOI:** 10.4274/tjo.galenos.2020.35305

**Published:** 2020-12-29

**Authors:** Gülşah Gümüş, Ahmet Kırgız, Yusuf Yıldırım, Nilay Kandemir Beşek, Selim Genç, Burçin Kepez Yıldız, Muhittin Taşkapılı

**Affiliations:** 1 University of Health Sciences Turkey İstanbul Beyoğlu Ophthalmology Training and Research Hospital, İstanbul, Turkey

**Keywords:** Infectious keratitis, penetrating keratoplasty, graft survival

## Abstract

**Objectives:**

To evaluate the prognostic factors affecting graft survival in patients undergoing penetrating keratoplasty (PKP) for infectious keratitis.

**Materials and Methods:**

Patients who underwent PKP for keratitis in our hospital between 2013 and 2018 were retrospectively reviewed. Patients who underwent therapeutic PKP at the inflammatory stage and were followed for at least 12 months were included in the study. Age, gender, follow-up period, time between diagnosis and surgery, lens status, presence of limbal involvement, presence of corneal ulceration, perforation, or corneal abscess, type of microorganism detected in culture, number of fortified medications used before surgery and duration of use, preoperative and postoperative visual acuity, postoperative graft transparency, postoperative complications, recurrence of infection, rate of re-keratoplasty, and indication for and timing of re-keratoplasty were recorded. The relationship between these findings and anatomic, therapeutic, and functional success were evaluated.

**Results:**

Fifty-nine patients were included in the study; 40 (67.8%) were male and 19 (32.2%) were female, and the mean age was 59.78±19.46 (6-91) years. Anatomic success was achieved in 58 patients (98.3%). Therapeutic success was achieved in 47 patients (79.7%) and there was a significant relationship between therapeutic success and re-keratoplasty and early re-keratoplasty (p<0.001 for both). Thirty-two patients (54.2%) had functional success and there was a significant relationship between the absence of postoperative complications and functional success (p=0.014).

**Conclusion:**

PKP is an effective treatment option in treatment-resistant keratitis or keratitis with impending perforation. The absence of postoperative complications and performing early re-keratoplasty in patients with recurrence increase the success rate.

## Introduction

Infective keratitis is a common sight-threatening condition worldwide.^1^ Between 1.5 and 2 million new cases of blindness associated with keratitis are reported each year in developing countries.^2,3^ Even with appropriate treatment, stromal abscess, severe corneal ulceration, descemetocele, and perforation can occur in some cases.^4^ Depending on the clinical presentation, available treatment options for patients with perforation include amniotic membrane transplantation, conjunctival flap cover surgery, repair with tissue adhesive, and therapeutic lamellar or penetrating keratoplasty (PKP).^5,6,7,8^

In infective keratitis, PKP can be performed for tectonic purposes to preserve globe integrity in patients with infectious perforation or suspected perforation, for therapeutic purposes to control infection in patients with uncontrolled infection, or for visual rehabilitation purposes in the late stage. PKP is known to yield more successful outcomes if performed at a later stage, after inflammation has regressed.^9^ However, cases with uncontrolled infection, perforation, or high risk of perforation may require PKP without waiting for inflammation to regress. Previous studies have evaluated the effects of patient age, sex, contact lens use, presence of systemic disease, history of trauma, whether keratitis was in the inflammatory stage, degree of corneal vascularization, graft diameter, ulcer and perforation size, type of microorganism detected in culture, postoperative complications, pre- and postoperative visual acuity, time elapsed between diagnosis and surgery, intraocular pressure (IOP), and lens status on graft survival in patients undergoing therapeutic PKP for keratitis.^9,10,11,12^ However, there has been no investigation into the impact of factors such as additional procedures performed concurrently with PKP, presence of corneal abscess, number of fortified drugs used preoperatively, whether re-keratoplasty was performed, and the indication and timing of re-keratoplasty.

The aim of this retrospective study was to evaluate the prognostic factors that affect graft survival in patients who underwent therapeutic PKP in our clinic due to infective keratitis.

## Materials and Methods

Patients who underwent therapeutic PKP due to keratitis in our clinic between 2013 and 2018 were retrospectively reviewed. Cases who underwent therapeutic keratoplasty in the inflammatory stage and were followed for at least 12 months were included in the study. Patients who did not have active infection, underwent refractive PKP, had suspected endophthalmitis in addition to keratitis, or were followed for less than 12 months were excluded. The study adhered to the principles of the Helsinki Declaration of Human Rights and received ethics committee approval. Consent was obtained from all patients for the use of their medical records.

The patients’ age, sex, duration of follow-up, time from diagnosis to surgery, history of contact lens use, presence of limbal involvement, corneal ulceration, perforation, or abscess, type of microorganism detected in culture, number of fortified drugs used preoperatively, PKP indication, additional procedures performed concurrently with PKP, graft diameter, pre- and postoperative visual acuity, postoperative graft transparency, postoperative complications, recurrence of infection, whether re-keratoplasty was performed, and the indication and timing of re-keratoplasty were recorded. Recurrence of infection was diagnosed upon repeated detection of infiltration by a microorganism previously detected on the graft at any time after PKP or based on the clinical presentation in cases with negative culture.

Pre- and postoperative visual acuity was measured as light perception, hand motions, counting fingers, or for longer distances, using Snellen chart and expressed in decimal. For microbiological diagnosis, corneal swab was collected from the site of infection. Samples were sent to the microbiology laboratory for direct examination, culture, and antimicrobial susceptibility testing. In case of suspected viral infection, corneal material was sent for polymerase chain reaction (PCR) analysis. Patients with suspected bacterial etiology were empirically treated with 50 mg/mL topical fortified vancomycin (Vancotek, Koçak, İstanbul, Turkey) and amikacin (Amikozit, Sanofi, Vilnius, Lithuania) or 50 mg/mL ceftazidime (İesetum, İbrahim Etem Ulagay, İstanbul, Turkey) drops (1 drop hourly) until a microbiological diagnosis was determined. Patients with suspected fungal keratitis were treated with 0.5 mg/mL fortified amphotericin B (Ambisome, Gilead Sciences, California, USA) or 10 mg/mL fortified voriconazole (Vfend, Pfizer, New York, USA) (one drop hourly) and 200 mg oral fluconazole (Fluzamed, World Medicine, İstanbul, Turkey) twice a day or 200 mg oral voriconazole (Vfend, Pfizer, New York, USA) twice a day. In addition, patients with suspected herpes simplex virus (HSV) infection were given 1.5 mg/g topical gancyclovir (Virgan, Thea, İstanbul, Turkey) 5 times a day and 800 mg oral acyclovir (Aklovir, Sandoz, Holzkirchen, Germany) 3 times a day. Empirical therapies were modified according to microbiologic and antimicrobial sensitivity results.

PKP outcome was evaluated separately as anatomic, therapeutic, and functional success. Anatomic success was defined as preservation of globe integrity and prevention of progression to phthisis bulbi. Therapeutic success was defined as the complete elimination of primary infection following PKP. Findings of corneal or scleral infiltrate, vitritis, or endophthalmitis were considered therapeutic failure. Functional success was defined as a postoperative gain in visual acuity compared to preoperative level.

Prognostic factors such as patient age, sex, time from diagnosis to surgery, lens status, presence of limbal involvement, corneal ulceration, perforation, or abscess, type of microorganism detected in culture, number of fortified drugs used preoperatively, PKP indication, additional procedures performed concurrently with PKP, graft diameter, preoperative visual acuity, postoperative complications, whether re-keratoplasty was performed, and the indication and timing of re-keratoplasty were evaluated in terms of their effects on anatomic, functional, and therapeutic success. Number of fortified drugs used before PKP was classified as ≤2 drugs or >2 drugs. Indication for PKP was categorized as treatment non-response or infective complications such as corneal ulcer, abscess, and perforation. Other procedures performed concurrently with PKP were grouped as lensectomy, vitrectomy, intraocular lens (IOL) extraction, intrastromal injection, subconjunctival injection, and intravitreal injection. Graft diameter was classified as <8.00 mm and ≥8.00 mm. Postoperative complications were noted as postoperative glaucoma, persistent epithelial defect, graft failure, cataract, endophthalmitis, phthisis bulbi, and retinal detachment. Indications for re-keratoplasty were grouped as graft rejection and recurrence of infection. The timing of re-keratoplasty was classified as <20 days or ≥20 days.

### Surgical Technique

All operations were performed under general anesthesia, retrobulbar anesthesia, or sub-Tenon’s anesthesia. Trephination extending 0.5 mm beyond the infected area was performed. After partial-thickness trephination of the recipient cornea, an anterior chamber incision was made with a 15-degree blade and the anterior chamber was filled with viscoelastic. The remaining corneal areas then were dissected using scissors. After removing the infected cornea, the anterior chamber was irrigated and any pupillary membrane, hypopyon, or fibrotic materials were cleared and anterior and posterior synechiae were released. The anterior chamber was washed with 1% vancomycin (Vancotek, Koçak, İstanbul, Turkey) and 2% ceftazidime (Iesetum, I.E. Ulagay, İstanbul, Turkey) in patients with anterior chamber bacterial keratitis and with 0.005% amphotericin B (Ambisome; Gilead Sciences, California, USA) or 1% voriconazole (Vfend, Pfizer, New York, USA) in patients with fungal keratitis until the corneal graft was placed. When required, the phakic lens or IOL was removed and anterior vitrectomy was performed. The donor cornea was cut from the endothelial side using a punch 0.25 mm larger than the trephine used. The donor cornea was sutured to the recipient bed using 10-0 nylon sutures. The operation was concluded with an intracameral injection of 2% ceftazidime (Iesetum, I.E. Ulagay, İstanbul, Turkey) or 1% voriconazole (Vfend, Pfizer, New York, USA) as an antifungal agent.

### Postoperative Treatment

Topical antimicrobial therapy was continued for at least 4 weeks in cases of bacterial keratitis and at least 12 weeks in cases of fungal keratitis. For patients with bacterial keratitis, topical 0.1% dexamethasone (Dexa-Sine SE, Liba, İstanbul, Turkey) or 1% prednisolone (Pred Forte, Allergan, Dublin, Ireland) was initiated at 6 times a day and was tapered to discontinuation at 12 months. For patients with fungal keratitis, 0.5% topical cyclosporine (Restasis, Allergan, Dublin, Ireland) was initiated for the first 2 weeks and if no recurrence of keratitis was observed, 0.1% dexamethasone or 1% prednisolone twice a day was added to the treatment after 2 weeks and was tapered to discontinuation at 12 months. Patients with herpetic keratitis received oral acyclovir (Aklovir, Sandoz, Holzkirchen, Germany) 800 mg 3 times a day for the first 4 weeks postoperatively and continued at a dose of 800 mg for at least 1 year. Artificial tears were prescribed to all patients. Antiglaucoma therapy was initiated if needed. Loose sutures were removed immediately.

### Statistical Analysis

Statistical analyses of the data were done using SPSS version 20.0 (IBM Corp., Armonk, NY) statistical package software. Statistical data were expressed as mean ± standard deviation. Descriptive statistics were expressed as frequency and percentage. Relationships between categorical variables and PKP success (anatomic, functional, therapeutic) were evaluated using chi-square test (Pearson or Fisher’s exact). For all analyses, the statistical significance level was accepted as p<0.05.

## Results

Of the 59 patients included in the study, 40 (67.8%) were male and 19 (32.2%) were female. The mean age was 59.78±19.46 (6-91) years. The mean follow-up time was 30.78±17.4 (12-72) months.

The patients’ preoperative characteristics and surgical indications are summarized in Table 1. The mean time from symptom onset to surgery was 18.68±15.2 (3-66) days. Of the 28 patients who underwent surgery within 10 days of symptom onset, PKP indication was keratitis-related complications (corneal abscess, perforation, ulceration) in 18 patients (64.29%) and non-response to treatment in 10 patients (35.71%) (p=0.01). The number of fortified drugs used before PKP varied between 1 and 4 (mean: 1.93±0.72). In the same session as PKP, concurrent lensectomy was performed in 6 patients, anterior vitrectomy in 1 patient, intrastromal injection in 1 patient, IOL removal in 1 patient, subconjunctival antibiotic injection in 3 patients, and 2 patients with intense anterior chamber reaction and hypopyon that underwent lensectomy received prophylactic intravitreal 1 mg/0.1 mL vancomycin (Vancotek, Koçak) and 2 mg/0.1 mL ceftazidime (Iesetum, IE Ulagay) because the posterior capsule was opened during lensectomy. Graft diameter ranged from 7.25 to 8.50 (mean: 7.76±0.31) and was ≥8.00 mm in 24 patients (40.68%) and <8.00 in 35 patients (59.32%).

One patient with no light perception underwent keratoplasty due to perforation and their postoperative vision was still at the level of no light perception. Preoperative visual acuity in the other patients ranged from light perception to 0.1 and their postoperative best corrected visual acuity ranged from light perception to 0.7 (Table 2). Thirty-one patients (52.54%) had postoperative visual acuity of light perception or hand motions, which was significantly reduced from the preoperative number of 49 patients (83.05%) (p=0.007). Postoperative visual acuity was 0.05 or better in 15 patients (25.42%), which was a significant increase from the 3 patients (5.08%) with that level of vision preoperatively (p=0.004). Seven patients (11.86%) had visual acuity of 0.1 or better at final examination.

Five (8.47%) of the patients had a history of contact lens use, 17 (28.81%) had trauma history, and 9 (15.25%) had a recent surgical history, while no etiology could be determined for 28 patients (47.46%). Trauma etiology was organic in 12 (70.59%) of the patients with trauma history. In microbiological examinations, bacteria were detected in 20 patients (33.90%), fungi in 12 patients (20.34%), and viruses in 5 patients (8.47%) as keratitis agents. Bacteriologic cultures yielded Staphylococcus aureus in 6 patients, Streptococcus pneumonia in 5 patients, Viridans streptococci in 3 patients, and Pseudomonas aeruginosa in 3 patients. In 1 patient, gram-negative bacillus was detected on direct examination but culture was negative. Fungal culture yielded Fusarium in 5 patients, Candida in 3 patients, and Aspergillus in 3 patients. In 1 patient, fungal hyphae were observed on direct examination but fungal culture was negative. PCR analysis for patients with suspected viral keratitis revealed HSV in 5 patients.

One patient (1.7%) who developed postoperative endophthalmitis was successfully treated with intravitreal antibiotic therapy. Retinal detachment occurred in 1 patient (1.7%) and pars plana vitrectomy was performed. Nine patients (15.3%) who developed cataract underwent phacoemulsification and IOL implantation surgery after achieving infection control. Twelve patients (20.3%) with elevated IOP responded well to medical treatment and none required glaucoma surgery. In 3 patients (5.1%) with refractory persistent epithelial defects, amniotic membrane transplantation resulted in epithelial healing. One patient (1.7%) with no light perception before PKP developed phthisis bulbi postoperatively. Three patients (5.08%) with limbal involvement received subconjunctival injection of fortified antibiotic. Despite no signs of recurrence, graft failure occurred in these patients. A total of 4 patients (23.7%) had graft failure. No complications were observed in 18 patients (32.2%).

At final examination, a clear graft was observed in 31 patients (52.5%), while 28 patients (47.5%) showed varying degrees of loss of graft transparency. Thirteen patients (22.03%) underwent re-keratoplasty. Indication for re-keratoplasty was graft rejection in 3 patients (23.07%) and recurrent infection in 10 patients (76.92%). Except for the patient with phthisis bulbi, anatomic success was achieved in the other 58 patients (98.3%) patients.

Therapeutic success was achieved in 47 patients (79.7%). Recurrent infection was observed in 12 patients (20.33%). Of these patients, 10 (83.33%) underwent re-keratoplasty, while in the other 2 patients (16.66%) the recurrent infection was controlled with antibiotic therapy. The mean time to detection of reinfection was 179±267.87 (3-720) days. Infection recurrence occurred within the first month in 6 patients (10.17%), within 1-3 months in 4 patients (6.78%), and between 3 months and 2 years in 2 patients (3.39%). The mean time between diagnosis and re-keratoplasty was 12.6±8.47 (2-26) days. Re-keratoplasty was performed earlier than day 20 in 8 patients (80%) and at day 20 or later in 2 patients (20%) with recurrent infection. Undergoing re-keratoplasty and early re-keratoplasty were significantly associated with therapeutic success (p<0.001 for both). Therapeutic success was not associated with patient age, sex, time from diagnosis to surgery, lens status, presence of limbal involvement, corneal ulceration, perforation, or abscess, type of microorganism detected in culture, number of fortified drugs used preoperatively, PKP indication, procedures performed concurrently with PKP, graft diameter, preoperative visual acuity, postoperative complications, or re-keratoplasty indication (p>0.05 for all) (Table 3).

Functional success was achieved in 32 patients (54.2%). The absence of postoperative complications was significantly associated with functional success (p=0.014). Functional success was not associated with patient age, sex, time from diagnosis to surgery, lens status, presence of limbal involvement, corneal ulceration, perforation, or abscess, type of microorganism detected in culture, number of fortified drugs used preoperatively, PKP indication, concurrent procedures, graft diameter, preoperative visual acuity, undergoing re-keratoplasty, or re-keratoplasty indication and timing (p>0.05 for all) (Table 3).

## Discussion

Patients with refractory keratitis are at risk of perforation, endophthalmitis, panophthalmitis, and even loss of the eye. Therapeutic PKP helps to eliminate the microorganism from the environment and to ensure tissue survival in cases of resistant keratitis. In these cases, successful PKP eradicates infection and ensures preservation of anatomic integrity and function of the eye.^13^ In the literature, anatomic success rates of 85.96% and 89.7% were reported by Raj et al.^11^ and Sharma et al.^10^, therapeutic success rates of 89.47%, 89.7%, and 97.6% were reported by Raj et al.^11^, Sharma et al.^10^, and Doğan and Arslan^14^, respectively, and Raj et al.^11^ reported 70.17% functional success in patients who underwent PKP due to keratitis. In our series of patients who underwent therapeutic PKP due to infectious keratitis, we achieved 98.3% anatomic success, 79.7% therapeutic success, and 54.2% functional success.

In their study on keratitis patients requiring inpatient treatment, Akova Budak et al.^15^ reported history of surgery in 10%, trauma in 10%, and contact lens use in 5% of patients and concluded that contact lens use and history of surgery and trauma were the most commonly identified etiologies in keratitis requiring inpatient treatment. Miedziak et al.^16^ reported that 3.3% of keratoplasty cases requiring PKP had a history of trauma, 8% had a history of contact lens use, and 46.7% had a history of surgery. Sharma et al.^10^ reported that although no etiology could be determined in 55.3% of cases, 33.2% of the patients had a history of trauma and that 54.7% of patients with trauma history had organic trauma. In our study, 8.47% of the patients had a history of contact lens use, 28.81% of patients had a history of trauma (70.59% of which were organic), and 15.25% of the patients had a history of recent surgery. The rate of organic trauma was higher in our study when compared with the literature.

In microbiological analysis of corneal samples from the patients in our study, bacteria were detected in 20 patients (33.90%), fungi in 12 patients (20.34%), and viral agents in 5 patients (8.47%), while no microorganisms were detected in culture or direct examination in 22 cases (37.29%). Doğan and Arslan^14^ determined the causative factor to be bacterial in 69.7%, viral in 14%, fungal in 11.6%, and Acanthamoeba in 4.6% of keratitis patients who underwent PKP. Yılmaz et al.^17^ reported bacterial infection in 28.2% and fungal infection in 8.06% of keratitis cases followed in their clinic. Sharma et al.^10^ detected bacteria in 31%, fungi in 20.9%, multiple pathogens in 6.9%, viruses in 5.3%, and Acanthamoeba in 1.6% of their patients. There may be several reasons for the inability to detect a microbiological agent in 37.29% of the patients in our study. First, most of the keratitis patients who presented to our clinic were referred from other centers after starting antimicrobial and steroid treatments, which may have prevented the detection of microbiological agent in some cases. Second, since our center is a branch hospital without facilities for microbiological analyses, the loss of time during sample transport to another center might have resulted in the inability to detect the microbiological agent.

The anatomic success rate was 98.3% in our study, consistent with previous studies.^10,18,19,20,21^ Raj et al.^11^ reported an anatomic success rate of 85.96% and cited preoperative visual acuity, PKP indication, postoperative complications, and graft transparency factors that significantly affected anatomic success. Sharma et al.^10^ attained 89.7% anatomic success in their study, and although the rate of anatomic failure was high in PKP performed on perforated eyes, they stated that this was not statistically significant. In our study, anatomic failure was not observed except in a patient with no light perception preoperatively who underwent PKP due to the risk of perforation.

Previous studies reported rates of graft transparency between 23% and 84.6%.^22,23,24,25,26^ Doğan and Arslan^14^ reported graft transparency rates of 83.3% at 1-year follow-up and 71% at 2-year follow-up, and noted that graft transparency was lower with grafts that were larger than 8 mm and those that were close to the limbus. In our study, the graft transparency rate was 52.5% after a mean follow-up of 30 months, and our results were in concordance with the literature. We also observed that graft transparency decreased in cases with larger graft diameter, but the difference was not statistically significant (p=0.09).

Functional success was achieved in 54.2% of the patients and there was a significant association between the absence of postoperative complications and functional success. The most common postoperative complications in our patients was elevated IOP (20.3%) and graft failure (23.7%). Raj et al.^11^ reported that postoperative complications significantly affected anatomic, functional, and therapeutic success. Although Sharma et al.^10^ determined that postoperative visual acuity was better in patients with smaller grafts, we did not observe a significant relationship between graft diameter and functional success (p=0.393). In our study, 11.86% of the patients had a visual acuity of 0.1 or better at final examination, which is a low rate compared to those in previous studies.^27^ The high rate of organic trauma and contamination in our patients and delayed presentation to our center due to starting treatment at another center are possible explanations for the poor visual outcomes.

Our therapeutic success rate was 79.7%. Recurrent infection was observed in 20.33% of patients and occurred after a mean of 179 days. Recurrent infection was observed within the first month in 10.17%, within 1-3 months in 6.78%, and between 3 months and 2 years in 3.39% of the patients. In their study, Lomholt et al.^9^ observed recurrent infection in 11% of patients within the first year, 16% within the first 2 years, and 24% within the first 5 years after therapeutic PKP. Bates et al.^28^ reported recurrent infection within 1-10 months (mean 3 months) after PKP performed in patients with keratitis. The mean time from diagnosis to re-keratoplasty in our study was 12.6 days. We observed that performing re-keratoplasty and early re-keratoplasty were significantly associated with therapeutic success. This may be because the most common indication for re-keratoplasty in our study was recurrence of infection, and performing re-keratoplasty at an early stage facilitated the eradication of the recurrent infection. Koçluk and Sukgen^12^ reported that early PKP yielded better anatomic and therapeutic outcomes. Sharma et al.^10^ also stated that PKP performed at an early stage, before perforation and limbal involvement, provided better outcomes. The lower therapeutic success rate in our study may be attributed to the high prevalence of microbiologically detected fungus.

Sharma et al.^10^ reported that small-diameter grafts (<9 mm) were associated with greater anatomic and functional success and that rates of recurrent infection and postoperative glaucoma increased with larger graft diameter. Raj et al.^11^ determined that when grafts were classified as larger and smaller than 8 mm in diameter, graft size had no effect on anatomic, functional, or therapeutic success. We also observed no significant relationship between graft diameter and functional or therapeutic success in our study. The fact that we used grafts larger than 8.50 mm in diameter in our patients may have limited the impact of graft size. Similarly, we did not detect a significant association between functional or therapeutic success and additional procedures performed concurrently with PKP, presence of corneal abscess, or the number of fortified drugs used preoperatively.

### Study Limitations

Limitations of our study include the limited number of patients, the retrospective study design, and that most of our patients were referred to our hospital from another center after the initiation of antibiotic treatment, which resulted in negative cultures.

## Conclusion

In conclusion, due to its high anatomic, therapeutic, and functional success, PKP is an effective treatment option in keratitis patients with resistant infection or impending perforation. Knowledge of the factors associated with post-PKP success will guide treatment planning.

## Figures and Tables

**Table 1:**
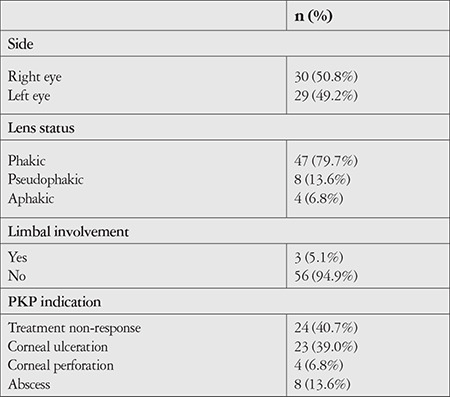
Preoperative data

**Table 2:**
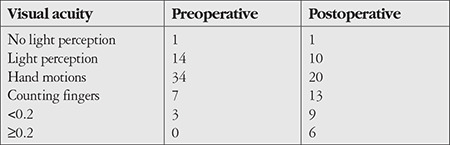
Pre- and postoperative visual acuity levels

**Table 3:**
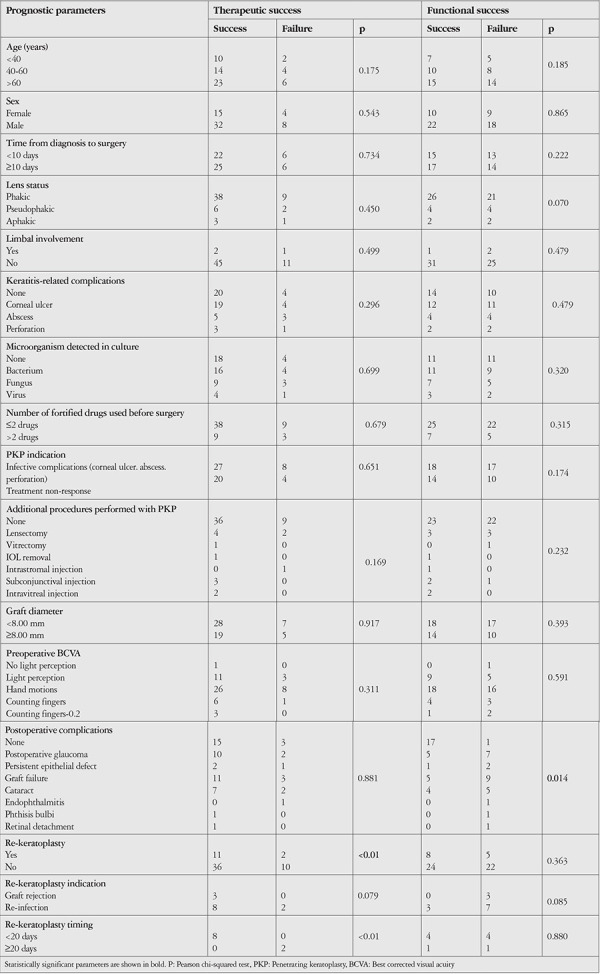
Relationships between prognostic factors and therapeutic and functional success
